# Case Report: Heterozygous *ADAR* c.3019G>A pathogenic variant associated with variable neurological symptoms and incomplete penetrance in a four-generational family

**DOI:** 10.3389/fimmu.2025.1453496

**Published:** 2025-07-18

**Authors:** Ann-Kathrin Bauer, Iris Marquardt, Benedikt Sundermann, Christine Wolf, Katrin Raupach, Kathrin Grundmann-Hauser, Laura Gieldon, Maximilian Otterbach, Martin Maurer, Tobias Haack, Min Ae Lee-Kirsch, Georg-Christoph Korenke, Marc-Phillip Hitz

**Affiliations:** ^1^ Institute of Medical Genetics, Carl von Ossietzky Universität Oldenburg, Oldenburg, Germany; ^2^ Department of Neuropediatrics, University Children’s Hospital, Klinikum Oldenburg, Oldenburg, Germany; ^3^ Institute of Radiology and Neuroradiology, Evangelisches Krankenhaus Oldenburg, Carl von Ossietzky Universität Oldenburg, Oldenburg, Germany; ^4^ Department of Pediatrics, Medizinische Fakultät Carl Gustav Carus, Technische Universität Dresden, Dresden, Germany; ^5^ Institute of Human Genetics, University Hospital Tübingen, Tübingen, Germany; ^6^ Institute of Diagnostic and Interventional Radiology, Klinikum Oldenburg, Carl von Ossietzky Universität Oldenburg, Oldenburg, Germany

**Keywords:** ADAR, c.3019G>A, Aicardi-Goutières syndrome, interferon signature, dystonia, spasticity, reduced penetrance

## Abstract

Heterozygous pathogenic variants in *ADAR* have been associated with dyschromatosis symmetrica hereditaria, while biallelic pathogenic variants have been associated with Aicardi-Goutières syndrome 6 (AGS6). However, the heterozygous variant c.3019G>A, (p.Gly1007Arg) has been described to cause neurological manifestations, which resemble AGS6 and are associated with an upregulation of interferon-stimulated genes. We report a four-generation family with two symptomatic family members and five unaffected carriers of the heterozygous pathogenic *ADAR* variant c.3019G>A. The index (patient 1) manifested a gait disorder at three years of age (weakness in his legs, a tendency to fall and hyperreflexia), dyslalia, and mild cognitive developmental delay. A paternal half-brother (patient 4) to patient´s father (patient 2) presented with irritability and regression of previous skills at the age of 6 months after a fever reaction, following the second routine hexavalent vaccination at the age of 4 months. At 20 years of age, the patient was wheelchair-bound, had spasticity and severe global development delay. A blood test in both patients showed increased interferon signature with activation of type 1-interferon. Five asymptomatic carriers were identified in this family (age range 2–81 years of age) nearly all of them (except the 81-year old patient) showed a strong activation of type 1 interferon response in peripheral blood. Affected individuals had higher interferon signature than asymptomatic, underlining the possible role of interferon activation in disease mechanism. To our knowledge, this is the biggest family reported to date, encompassing a wide age-range of carriers, including an asymptotic carrier of advanced age (81 years of age).

## Introduction

The adenosine deaminase acting on RNA gene (*ADAR*) codes for an enzyme that catalyses the conversion of adenosines to inosines in double-stranded RNA and thereby renders self RNA invisible to nucleic acid sensing receptors of the innate immune system (1). A reduced or absent ADAR activity is associated with an activation of interferon-stimulated genes due to aberrant sensing of self RNA (2, 3).

Heterozygous pathogenic variants in *ADAR* have been associated with dyschromatosis symmetrica hereditaria (DSH) and biallelic pathogenic variants have been associated with Aicardi-Goutières syndrome 6 (AGS6) (4, 5). DSH manifests with hyper- and hypopigmented macules on the dorsal aspect of hands and feet that appear in infancy or early childhood (5). Additionally, freckle-like macules are seen on the face. These macules are usually asymptomatic (5).

AGS is a type-1 interferonopathy that presents with variable neurological symptoms, mainly spasticity, dystonia, leukoencephalopathy associated with variable degrees of developmental delay and intellectual and physical disability (4). Onset has been described ranging from infancy to later childhood. Mostly, affected children develop age-appropriately before onset of disease. In some children, symptoms rapidly progress with a regression of previously acquired skills. Furthermore, cardiac valve involvement has been reported (6–8). Cranial MRI findings in *ADAR*-associated AGS include encephalopathy, brain atrophy, calcifications, bilateral striatal necrosis and globus pallidus signal alterations (4, 6, 8–11). Furthermore, AGS has been described as a type-1 interferonopathy with an up-regulation of type 1 interferon signalling, measured by the expression of interferon-stimulated genes (ISGs) (3, 4, 8).

The *ADAR* variant c.3019G>A, has been described to cause neurological manifestations, resembling AGS also in a heterozygous state (9). The variant has been shown to have a significant effect on editing, which is comparable to the inactive protein. It is assumed to exert a dominant negative effect by being catalytically inactive and competitively inhibiting the wildtype protein (4). Ultimately, the transcription of interferon stimulated genes (ISGs) is activated (3). To date, only few affected individuals have been described in literature. Clinical manifestation was very variable, presenting with or without DSH, spasticity, dystonia, rapid psychomotor regression and cognitive decline (6, 9, 12). In some patients, aortic valve sclerosis has been described (13). Furthermore, an association with upregulation of ISGs has been reported (6, 9). Symptoms are thought to be triggered by an infection, fever and possibly a vaccination in previously healthy children (6, 8).

Here we report a four-generational, non-consanguineous family with multiple carriers of the heterozygous pathogenic *ADAR* variant c.3019G>A with two affected and multiple asymptomatic individuals, highlighting incomplete penetrance and variability of neurological symptoms.

## Methods

The index, patient 1, presented to our departments of neuropediatrics and medical genetics. Genome sequencing was performed in a diagnostic setting at the Institute of Medical Genetics and Applied Genomics (IMGAG) in Tuebingen using blood-derived genomic DNA. Library preparation was conducted using the TruSeq PCR-free library prep kit from Illumina. Paired-end sequencing (2x150 base pairs) was performed on an Illumina NovaSeq6000 sequencer. The sequence data was analysed using the megSAP pipeline (https://github.com/imgag/megSAP) and aligned to the GRCh38 reference genome. Phenotype-based filtering for rare variants (minor allele frequency ≤1% in Genome Aggregation Database (gnomAD, gnomad.broadinstitute.org) and ≤ 5 occurrences in an IMGAG in-house database) prioritized the pathogenic *ADAR* variant. Subsequent carrier testing in the parents and additional family members was done by Sanger sequencing and data analysis using the SEQPilot software from JSI medical systems. For patient 4, genome sequencing was performed as outlined above to exclude possible other disease-causing variants.

Expression of the ISGs *IFI27, IFI44, IFI44L, IFIT1, ISG15, RSAD2* and *SIGLEC1*, normalized to GAPDH and HPRT1 in peripheral blood mononuclear cells were assessed. The relative gene expression (fold-change) was compared to the average gene expression of 10 healthy controls. Gene expression was analysed with quantitative RT-PCR (TaqMan) and calibrated. The mean values of at least three measurements were reported.

All medical information, genetic testing and further laboratory investigations were obtained in routine clinical care. Available clinical MRI data were re-assessed for findings typical of AGS. Written informed consent was obtained from each patient and/or their legal guardian.

## Case description

### Patient 1

**Figure 1:**
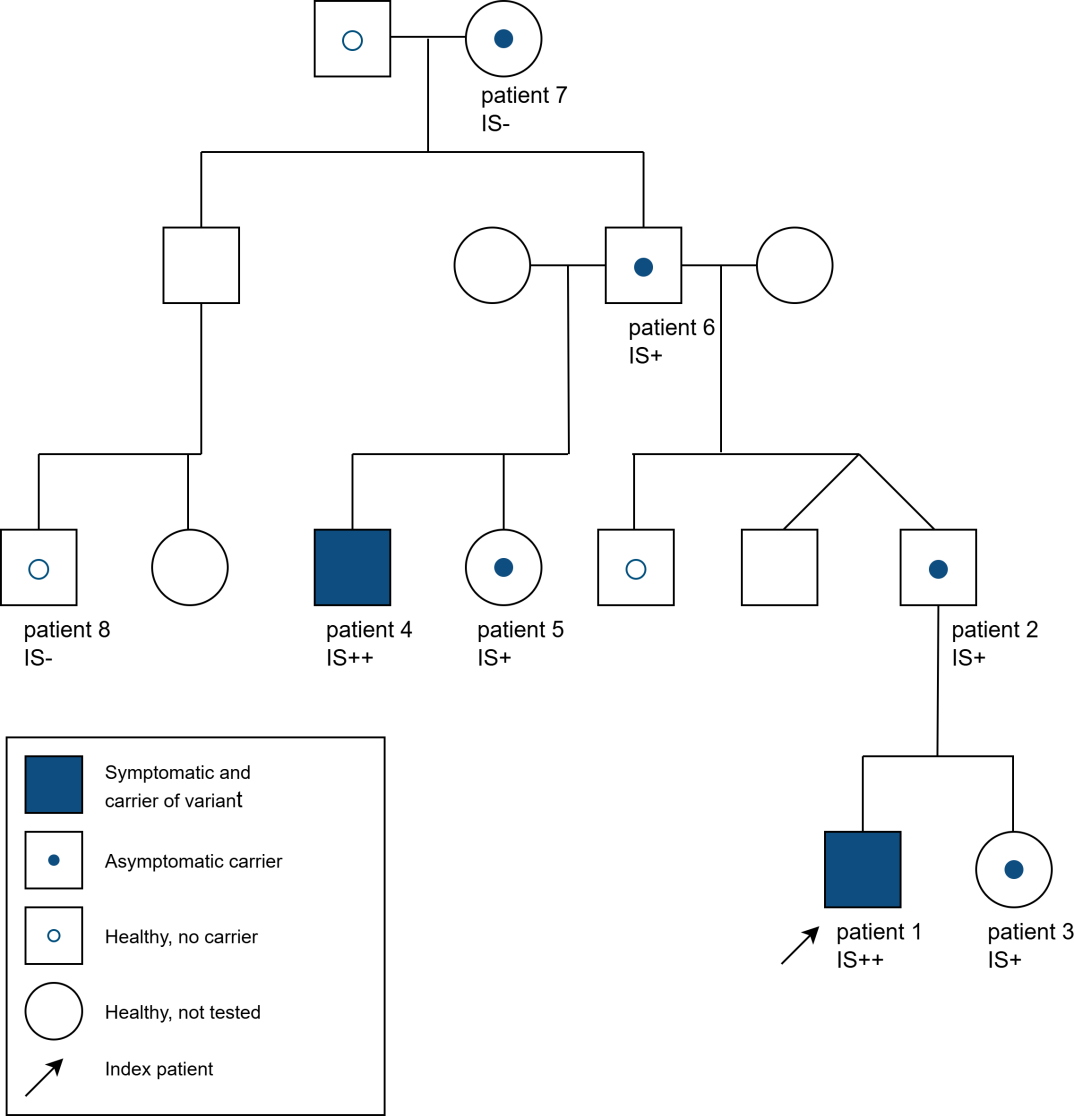
Pedigree of the family with various asymptomatic and symptomatic carriers of the heterozygous *ADAR* variant c.3019G>A and interferon signature (IS) in some family members [- = no activation, + = strong activation (score 60-200, normal value <12,49), ++ = very strong activation (score >200)].

**Figure 2:**
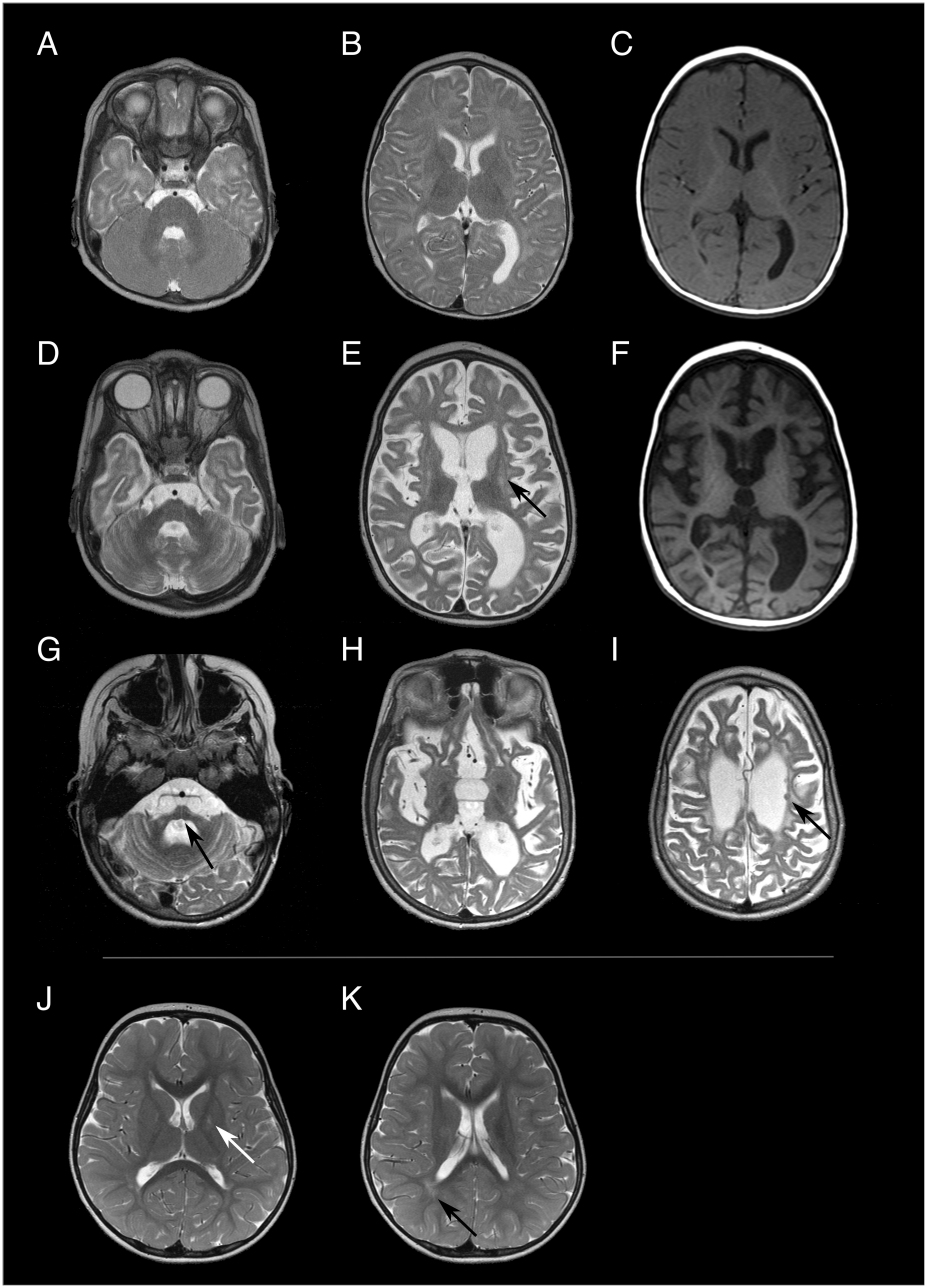
Brain MRI images of patient 4 [**(A-C)** at 8 months of age, **(D-F)** at 15 months of age, **(G-I)** at 10 years of age] and patient 1 [**(J, K)** at 3 years and 8 months of age]. Both patients exhibit different degrees and patterns of brain alterations. In the first MRI examination, patient 4 had a normal brain volume and the white matter signal was mainly compatible with delayed myelination both in T2w- **(A, B)** and T1-weighted **(C)** images. Only 8 months later signs of global atrophy were present, including volume loss and T2 hyperintensity of the striatum [arrow in **(E)** putamen]. Atrophy progressed to severe supra- and infratentorial volume loss [arrow in **(G)** pons] at 10 years of age. Few T2 hypointense nodular lesions were present in the periventricular white matter at all three time points [arrow in **(I)**], which could not be unambiguously identified as either focal calcifications or nodular heterotopia. Patient 1 only exhibited minor and relatively unspecific T2 hyperintense signal alteration in the globus pallidus [arrow in **(J)**] and the periventricular white matter [arrow in **(K)**] at 3 years and 8 months years of age.

The index patient (patient 1; **Figure 1**) developed age-appropriately until the age of three years. He then developed a gait disorder with weakness in his legs, a tendency to fall, dyslalia and mild cognitive developmental delay. Physical examination demonstrated hyperreflexia of lower extremities and bilateral Trendelenburg sign. The symptoms are consistent with AGS and in the AGS severity scale (14) he scored 10 points. No fever or infection preceded symptom development. Cerebral spinal fluid (CSF) neopterin was elevated (76 nmol/l, normal < 32 nmol/l). A cranial MRI showed minor signal elevation in T2-weighted images in the globus pallidus (**Figure 2J**) and periventricular white matter (**Figure 2K**), possibly consistent with myelination defects. MRI findings resembled previously reported alterations related to *ADAR* variants (6, 9). They were, however, subtle and thus relatively unspecific. There was no evidence of cerebral calcifications in susceptibility-weighted MRI images. Genome sequencing (GS) identified the heterozygous variant c.3019G>A, p.(Gly1007Arg) in *ADAR* ((ENST00000368474.9):c.[3019G>A];[=], p.[(Gly1007Arg)];[(=)]). No other possible disease-causing variants were identified. A blood test showed strong interferon signature with activation of type 1- interferon (multiple measurements, scores between 719 and 4623, normal value < 12,49). Additionally, increased expression of CD169+ and CD206+ was observed (see **Supplementary Table 1** for more information). Echocardiographical examination of the heart was normal. At four years of age, the patient’s symptoms showed slow progression with progressive gait disorder and progressive leg weakness. A nerve conduction velocity test showed beginning demyelinating polyneuropathy in his legs. Due to progressive symptoms, a treatment with ruxolitinib (dosis twice daily 12,5mg) was initiated. Previously, a treatment option with ruxolitinib, a selective Janus kinase inhibitor has been described as effective in *ADAR*- *TREX1*- and *IFIH1*- related AGS (9, 15, 16). At five years of age, after half a year of treatment with ruxolitinib, neurological symptoms are stable.

### Patients 2 and 3

The pathogenic *ADAR* variant was also identified in the index patient’s asymptomatic father (patient 2). Cranial MRI and neurological examination at 28 years of age had been reported as being normal (MRI images not available). Blood test showed an elevated interferon signature (score 110). The variant was also detected in the two years old sister of patient 1 (patient 3). She did not show any symptoms and EEG was age-appropriate. Multiple blood tests also showed activation of type 1 interferon (scores ranging from 83- 1026).

### Patient 4

A paternal half-brother (patient 4) to the father (patient 2) presented at approximately 6 months of age after a fever reaction following the second routine hexavalent vaccination two months before. He developed restlessness, irritability, opisthotonus, regression of previous skills and spasticity. Physical examination at nine months of age showed muscular hypertonia of the extremities, weak reflexes and spontaneous Babinski sign. Cerebral spinal fluid (CSF) neopterin was elevated (305 nmol/l and 501nmol/l at 11 and 18 month of age respectively). The initial cranial MRI at the age of 8 months (**Figures 2A–C**) showed a normal brain volume. At the age of eight months, white matter signal was consistent with moderately delayed myelination, but could not be fully differentiated from potential initial leukoencephalopathy. Follow-up MRI, at the age of 15 months, (**Figures 2D–F**) exhibited generalized T2 signal elevation of the white matter, T2 hyperintensities in the putamen, dentate nucleus (striatum) and globus pallidus associated with pronounced volume loss as well as generalized brain atrophy. Brain atrophy further progressed (**Figures 2G–I**). All three available MRI examinations showed minor nodular hypointense lesions in T2-weighted images, which could represent minor parenchymal calcifications, but which were ambiguous in the available MRI data. At 20 years of age, the patient was wheelchair-bound and had severe global development delay, tetraparesis with spasticity, multiple joint contractures and hip luxation, scoliosis and inadequate nutrition. The symptoms are consistent with AGS and in the AGS severity scale (14) he scored 0 points.

Blood tests also showed a strong activation of type 1 interferon (score 1114). The familial heterozygous *ADAR* c.3019G>A variant was detected by Sanger sequencing and whole-genome sequencing showed no other variant that could be associated with his symptoms.

### Patients 5, 6, 7 and 8

The sister (patient 5) of this second affected male (patient 4) in the family also carries the same *ADAR* variant. At 10 years of age, she did not show any symptoms. Measurement of type 1 interferon was not available.

The father of patient 2, 4 and 5 (patient 6) also carries the same pathogenic *ADAR* variant and shows strong activation of type 1 interferon (score 63) but was asymptomatic at age 59. The pathogenic *ADAR* variant was also detected in the mother of patient 6 (patient 7). At 81 years of age she shows no neurological symptoms and there was no detectable interferon signature (score <12,49). In a paternal cousin to patient 2 (patient 8) the heterozygous *ADAR* variant c.3019G>A was not detected and interferon measurements showed no detectable signature. To our knowledge, no one in the family showed any skin manifestations related to DSH. Patients 1 and 3 are of Caucasian-Turkish descent, the rest of the family are Caucasian. An overview of all available features of symptomatic and asymptomatic variant carriers is displayed in **Table 1**.

**Table 1:** Features of symptomatic and asymptomatic variant carriers in the family.

Patient	Age (in years)	Variant	Symptoms	Physical findings	MRI findings	Blood work	CSF neopterin levels	Co-morbidities	Immunizations
1	5	*ADAR* c.3019G>A het	Gait disorder, leg weakness, dyslalia, mild cognitive developmenal delay, AGS severity scale: 10 points	Hyperreflexia legs, bilateral Trendelenburg sign NCV: demyelinating polyneuropathy	Minor signal elevation in T2-weighted images in globus pallidus and periventricular white matter	Interferon signature multiple measurements scores between 719 and 4623. Bloodcount, Elektrolytes, CK- levels normal. See also supplement 1	76 nmol/l	None	Vaccinations following national immunization schedule by STIKO up until 4 years of age, thereafter only inactivated vaccinations.
2	28	*ADAR* c.3019G>A het	None	Neurological examination normal NCV: normal EEG: normal	Reported normal (images not available)	Interferon signature score 110	Not performed	None	Vaccinations according to national immunization schedule by STIKO
3	2	*ADAR* c.3019G>A het	None	Neurological examination normal EEG: normal	Not performed	Interferon signature multiple measurements scores between 83- 1026	Not performed	None	Vaccinations according to national immunization schedule by STIKO up until 10 months of age, thereafter only inactivated vaccinations.
4	20	*ADAR* c.3019G>A het	Restlessness, opisthotonus, regression of previous skills, spasticity, severe global developmental delay, tetraparesis, AGS severity scale: 0 points	At 9 m: muscular hypertonia of the extremities, weak reflexes spontaneous Babinski sign	15m: generalized T2 signal elevation of white matter, T2 hyperintensities in putamen, dentate nucleus (striatum) and globus pallidus, generalized brain atrophy. All images: hypointense lesions in T2-weighted images, which could represent minor parenchymal calcifications	Interferon signature score 1114	Age 11 m 305 nmol/l Age 18 m 501 nmol/l	None	Initially vaccinations according to national immunization schedule by STIKO up until second routine hexavalent vaccination.
5	10	*ADAR* c.3019G>A het	None	Normal	Not performed		Not performed	None	Only measles vaccination
6	59	*ADAR* c.3019G>A het	None	Normal	Not performed	Interferon signature score 63	Not performed	Myocardial infarct at age 52	Vaccinations according to national immunization schedule by STIKO
7	81	*ADAR* c.3019G>A het	None	Normal	Not performed	Interferon signature negative		Diabetes mellitus, Breast-cancer	Vaccinations according to national immunization schedule by STIKO
8	34	Wild type	None	Normal	Not performed	Interferon signature negative	Not performed	None	Vaccinations according to national immunization schedule by STIKO

NCV, nerve conduction velocity; STIKO, Standing Commission on Vaccination; MMR, Measles; Mumps and Rubella Vaccination.

## Discussion

We report a four generational family with multiple heterozygous carriers of the pathogenic *ADAR* variant c.3019G>A with two symptomatic family members and five unaffected carriers. Symptoms in the two affected individuals differed. While in patient 4, symptom onset was at around 6 months of age with rapid progression, patient 1 showed symptoms at 3 years of age and slow disease progression. Other family members were asymptomatic at various ages. Cranial MRI findings in both affected individuals were substantially different regarding both pattern and extent, with signal alterations in the globus pallidus being the only commonality. MRI findings were within the spectrum previously reported in *ADAR*-related AGS (6, 8, 9). Upregulation of type-1 interferon was shown in nearly all available family members (except patient 7), showing the highest elevation in the affected individuals. To our knowledge, the heterozygous *ADAR* pathogenic variant c.3019G>A has been described in 22 further cases (isolated and familiar), associated with AGS symptoms (6, 8, 9, 12, 13, 17–19).

Neurological symptoms described included dystonia, spasticity, tetraparesis, rigidity, irritability and regression of previous skills. Intellect was severely affected in some individuals and preserved in others. Age of onset (if known) spanned from 4–6 months to 17 years of age (8, 9, 13). This variability in symptom onset, disease progression, affected intellect and symptoms is also reflected in our family with the two affected individuals having variable disease manifestation. It has been reported, that symptoms developed after an infection and/or vaccination in previously age-appropriately developed children (8). This could also be observed in patient 4, where symptoms were thought to be triggered after a fever reaction, possibly in the context of a vaccination. For patient 1, no infect association was reported. Furthermore, DSH was present in the minority of described cases and not present in this family (9). Generally, DSH has rarely been described outside of Japan and China (4).

Inheritance of the *ADAR* c.3019G>A variant from asymptomatic or (mildly) symptomatic parents has been described in previous literature (6, 8, 12, 13). Paternal inheritance was assumed in two affected paternal half- sisters (father not available for testing). The father showed no *ADAR*-related symptoms (6). Kondo et al. described inheritance from a mother, who only presented with faint hypopigmented macules on the dorsal aspects of her fingers and no neurological symptoms (12). Rice et al. described subtle psychological features and basal ganglia calcification in a mother of an affected child (8). Furthermore, Tojo et al. described a father of an affected child harbouring the same pathogenic *ADAR* variant. He had DSH features from childhood on and in his early thirties he developed irritability, mental deterioration and severe aortic valve sclerosis (13). These examples show that later disease onset and significant intrafamilial variability is possible in *ADAR*-related disease (8). Additionally, Crow et al. described a 68-year-old asymptomatic individual with a homozygous pathogenic variants in *RNASEH2A*, associated with AGS (20). The family in this case report further undermines the strong intrafamilial variability and incomplete penetrance with the oldest family member showing no ADAR-related symptoms at the age of 81 years. Possible influencing factors could be genetic modifiers (protective or susceptibility alleles, epigenetic factors) and exposure to immunogens or other environmental triggers but exact pathomechanism and possible triggering factors warrant further research.

Previously, CSF neopterin has been reported as a valuable diagnostic tool in AGS patients (9). Elevated neopterin could also be observed in patient 1 and patient 4, underlining its possible diagnostic relevance.

In this family, nearly all carriers showed strong activation of type 1 interferon in blood. For patient 5 interferon signature was not available since the parents did not agree on conducting the measurement. This activation, however, was markedly stronger in affected individuals, highlighting the potential role as a disease biomarker and monitoring (8).

In prior literature, a mildly affected mother with *ADAR* c.3019G>A of a son with AGS related disease, showed an elevated interferon signature (scores between 25.743 and 12.836) (8). Some parental carriers of other recessive *ADAR* pathogenic variants (p.Pro193Ala and other) showed an elevated interferon signature (maximum score 22.5) (8). So far, no elevated interferon scores were reported from asymptomatic carriers of the *ADAR* c.3019G>A variant. We report four asymptomatic carriers with elevated interferon scores (score range 63 – 110), while the oldest carrier in this family did not present elevated scores. This is in accordance with the oldest carrier being asymptomatic and has been reported in other *RNASEH2A* –related AGS in an asymptomatic 68-year-old individual (20). However, we only conducted a single measurement and cannot exclude preanalytic or technical factors.

Cranial MRI showed signal alterations in the globus pallidus in both affected members of this family. While in patient 4 progressive cerebral atrophy could be seen, this was not detected in patient 1. Previously, MRI findings in affected individuals also detected changes in the basal ganglia with hypointensity of the striatum and pallidum (9). Furthermore, calcification and bilateral striatal necrosis have been reported in *ADAR*-related disease (6, 8). Generally, MRI findings tend to vary and some cases of normal cranial MRIs have been reported (8). In patient 1 bilateral striatal necrosis and calcification were not seen. In patient 4 possible calcifications could be seen. However, a CT-scan or susceptibility-weighted MRI images, where intracranial calcifications tend to be more visible, were not performed. In this family, available cranial MRI from one asymptomatic individual (patient 2) had been reported as a normal finding. Generally, limited information on MRI findings of asymptomatic carriers is available in literature. In one mildly affected parent described by Rice et al., 2017, white matter and cerebellar calcifications could be observed (8).

Patient 1 received ruxolitinib to halt disease progression. After half a year of treatment, his symptoms were stable. In previous literature treatment trials with levodopa, ruxolitinib and dexamethasone have been described (13). Treatment with levodopa has been described with variable efficacy (13). Treatment with ruxolitinib, a selective Janus kinase inhibitor, has been described as effective in some features of *ADAR*- *TREX1*- and *IFIH1*- related AGS by blocking interferon signalling (9, 15, 16). Especially treatment initiation in early stages of the disease has been considered important (15, 16).

## Conclusion

The heterozygous pathogenic variant *ADAR* c.3019G>A shows incomplete penetrance in this family and might be causative for variable neurological symptoms in two affected family members. Other families have been described in which symptomatic children inherited the same variant from asymptomatic or (mildly) symptomatic parents (6, 8, 13). To our knowledge, this is the biggest family reported to date with a wide age-range of carriers and an asymptomatic carrier of advanced age (81 years of age). Furthermore, interferon profiles from nearly all carriers (except patient 5) in the family were conducted and, except for one, all were elevated. Affected individuals had higher interferon signature than asymptomatic, underlining the possible role of interferon activation in disease mechanism. This high intrafamiliar variability illustrates the diagnostic challenges and possibly underrecognized individuals and warrants further research into disease mechanism. A treatment option to halt disease progression with prednisolone or selective Janus kinase inhibitors has been suggested (6, 15). This makes early identification of symptomatic variant carriers even more important.

## Data Availability

The datasets presented in this study can be found in online repositories. The names of the repository/repositories and accession number(s) can be found below: https://www.ncbi.nlm.nih.gov/clinvar/variation/39458/, VCV000039458.45.
