# Supplementary material for: Case Report: Heterozygous ADAR c.3019G>A pathogenic variant associated with variable neurological symptoms and incomplete penetrance in a four-generational family
Source: Front Immunol. 2025 Jul 18;16:1453496. doi: 10.3389/fimmu.2025.1453496 (PMC12313608; doi:10.3389/fimmu.2025.1453496)
Supplement: Supplementary file 1 [file Table1.docx]

**Supplementary Table 1:** Immunological testing of patient 1.

| **Measurement** | **Value** | | **Normal value** |
| --- | --- | --- | --- |
| CD169+ monocytes | 9681 antigens/cell | 10.910 antigens/cell | <1400 |
| CD206+ monocytes isotype | 391 antigens/cell | |  |
| Polio Type 1 Antibodies | 1:160 | | > 1:10 Immunity assumed |
| Polio Typ 3 Antibodies | 1:160 | | > 1:10 Immunity assumed |
| Pneumococcal IgG Antibodies | 32,8 mg/dl | | 15,4 -40,8 mg/dl at 4 years of age |
| Diphtheria Toxoid EIA | 0,29 IE/ml | | 0,10-1.00 Immunity assumed |
| Tetanus-Toxoid | 0,3 IE/ml | | 0,10-1.00 Immunity assumed |
| Varicella-Zoster Virus (VZV) IgG | 860 mU/ml | | < 150 mU/ml |
| Epstein-Barr Virus (EBV) EBNA IgG (Epstein-Barr Nuclear Antigen IgG) | 30 U/ml | | < 20 U/ml |
| Epstein-Barr Virus (EBV) VCA IgG (Viral Capsid Antigen IgG) | <10 U/ml | | < 20 U/ml |
| Mumps IgG | 112 AU/ml | | < 11 AU/ml |
| Measles IgG | 27.7 AU/ml | | < 16.5 AU/ml |
| Rubella IgG | 113 IU/ml | | < 10 IU/ml |
